# Supplementary material for: The Role of White Matter Disconnection in the Symptoms Relating to the Anarchic Hand Syndrome: A Single Case Study
Source: Brain Sci. 2021 May 14;11(5):632. doi: 10.3390/brainsci11050632 (PMC8156999; doi:10.3390/brainsci11050632)
Supplement: Supplementary file 1 [file brainsci-11-00632-s001.zip › brainsci-1165798-supplementary.pdf]

Supplementary Materials

The role of white matter disconnection in symptoms relating to the anarchic hand syndrome: a single case study.

Pacella Valentina, Ricciardi Giuseppe Kenneth, Bonadiman Silvia, Verzini Elisabetta, Faraoni Federica, Scandola Michele, Moro Valentina

A. REVISION OF LITERATURE REGARDING THE ANARCHIC HAND SYNDROME

A selection of studies was made by using Pubmed indexed articles published between 2016 and 2020 and following the criteria previously used in Moro et al., 2015. 'Anarchic hand', or 'alien hand' were used as the key terms. Articles written in languages other than English were not included. The search identified 23 potentially relevant articles, to which inclusion criteria were applied. The inclusion criteria were: i) the presence of at least one AHS case described in the paper; ii) a detailed neuropsychological assessment and iii) the presence of a localised brain lesion with a documented analysis of the lesion or the presence of at least four documented CT/MRI scan slices. The exclusion criteria were: i) AHS in degenerative syndromes or in developmental disorders and ii) the presence of only one unclear symptom (e.g. grasping, levitation).11 articles met all of the criteria and were included in the systematic review. The symptoms reported in these articles are summarised in table SM1.

Table SM1. Review of the literature on AHS from 2016 to 2020.

Legend. N: The symptom is reported as “not present”; nc: not conclusive, the symptom or deficit is not clearly specified or there are not enough elements to clearly state its presence; **C**, corpus callosum; **F,** frontal; **P**, parietal; **T**, temporal; **O**, occipital; **CgM**, Cingulate gyrus (motor region);**Thal**, thalamus; **nCh**: non-chronic; **ns,** not specified.

Purposeful or semi purposeful AH movements: **Mag apr**, Magnetic apraxia; **Grasping**, Grasp reflex; **Forced Grasp**, Forced grasping reflex; **Groping**, Groping reflex; Other, **UB**, Unilateral utilisation behaviour; Other, **CM**, Compulsive manipulation of tools. *Non purposeful AH movements:* **Expl beh**, Exploratory behaviour; **Rep mov**, Repetitive movements; **Self grabbing**; **Levitation**; **Noct mov**: AH nocturnal movements. *Uncontrolled bilateral hand movements:* **Int conf**, Intermanual conflict; **Diag Dysp**, Diagonistic Dyspraxia; **Responsiveness**; **Mirr**, AH mirror movements; **SinK**, Synkinesias. *Feelings:* **Alien**, Alien hand; **Per**, personification; **Rest**, restraining actions (**R**); Aut, autocriticism (**A**); **Avoi**, avoidance behaviour (**V**). *Disconnection symptoms:* **Unil apr/agr**: unilateral apraxia (**Ua**) / agraphia (**Ug**); **Tact agn**, unilateral tactile Agnosia; **Int tran**: Deficits in interhemispheric sensory transmission. *Frequently associated sensorimotor deficits:* **Bi Inc**, bimanual incoordination; **Tap**, tapping deficit; **Seq**, action sequence deficit; **Sensory loss**, AH sensory loss; **Lower limb dis**, Lower limb sensory loss or hyposthenia; **Mutism** (initial), Akinetic mutism in the acute phase.

Cognitive deficits: **Language dis**, language disorders. **Verbal mem**, verbal memory; **Vis mem**, Visual memory deficits (**Vi**); **Cons**: constructional apraxia deficit (**Co**); **Attentional dis**, attentional disorders; **Disexecutive synd**: disexecutive syndrome.

References (articles from 2016 to 2020): 1= Gheewala et al., 2019; 2= Murdoch et al., 2021; 3= Demiryurek et al., 2016; 4= Russo et al., 2020; 5= Nowak et al., 2020; 6= Alvarez et al., 2020; 7= Gao Xiaove et al., 2016; 8= Khalighinejad et al., 2017; 9= Mahawish, 2016; 10= Qureshi et al., 2016; 11= Abdulla and Saseendran, 2019

*Total numbers of symptoms**		**Posterior, Right AH**		**Posterior, Left AH**			**Callosal, Right AH**		**Callosal, Left AH**		**Mixed, Right AH**	**Mixed, Left AH**
Reference		1	2	3	4	5	6	7	8	9	10	11
Total Patients		2		3			2		2		1	1
Callosal and Cortical Lesions		O T P	P O	P	N	O T P	C	C	C F	C	C P	C
Subcortical Lesion		ns	ns	ns	Thal	Thal	ns	ns	CgM	ns	ns	CgM
AH duration		ns	ns	ns	ns	ns	ns	ns	ns	ns	nCh	nCh
Purposeful AH movements	Mag apr											
	Grasping								1			
	Forced Grasp								1			
	Groping								1		1	
	Other								1			
Non purposeful AH movements	Expl beh											
	Rep mov											
	Self-grabbing	1		1							1	
	Levitation			2								
	Noct mov											
Uncontrolled bilateral hand movements	Int conf			1			2		1		1	1
	Diag Dyspraxia						1		1			1
	Responsiveness											
	Mirr, Sink											
Feelings	Alien, Pers			1			1				1	
	Rest, Aut, Avo	1		3			1					
Disconnection symtomps	Unil apr, agr											
	Tact agn, Int tran											
Frequently associated sensorymotor deficits	Unresponsiveness								1			
	Dexterity											
	Bi Inc, Tap, Seq	1							1			
	Sensory loss			2								
	Lower limb hyp											
	Mutism (initial)											
Cognitive deficits	Language dis											
	Verbal mem			1								
	Vis mem, Cons											
	Discalculia											
	Attentional dis			1					1			
	Neglect	1		1								
	Disexecutive synd											
	Apraxia						1				1	1

*In the review of the literature 2016-2020 and considering the inclusion criteria, no cases of AHS due to anterior lesion were found.

1. Gheewala, G.; Gadhia, R.; Surani, S.; Ratnani, I. Posterior Alien Hand Syndrome from Acute Ischemic Left Parietal Lobe Infarction. *Cureus* **2019**, doi:10.7759/cureus.5828.
2. Murdoch, M.; Hill, J.; Barber, M. Strangled by Dr Strangelove? Anarchic hand following a posterior cerebral artery territory ischemic stroke. *Age Ageing* **2021**, *50*, 263–264, doi:10.1093/ageing/afaa129.
3. Demiryürek, B.E.; Gündogdu, A.A.; Acar, B.A.; Alagoz, A.N. Paroxysmal posterior variant alien hand syndrome associated with parietal lobe infarction: case presentation. *Cogn. Neurodyn.* **2016**, *10*, 453–455, doi:10.1007/s11571-016-9388-y.
4. Russo, M.; Carrarini, C.; Dono, F.; Di Stefano, V.; De Angelis, M.V.; Onofrj, M.; Sensi, S.L. Posterior Variant of Alien Limb Syndrome with Sudden Clinical Onset as Self-Hitting Associated with Thalamic Stroke. *Case Rep. Neurol.* **2020**, *12*, 35–39, doi:10.1159/000503857.
5. Nowak, D.A.; Engel, A.; Leutbecher, M.; Zeller, C. Alien limb phenomenon following posterior cerebral artery stroke: a distinct clinical entity. *J. Neurol.* **2020**, *267*, 95–99, doi:10.1007/s00415-019-09543-2.
6. Alvarez, A.; Weaver, M.; Alvarez, G. Rehabilitation of Alien Hand Syndrome Complicated by Contralateral Limb Apraxia. *Am. J. Phys. Med. Rehabil.* **2020**, *99*, e122–e124, doi:10.1097/PHM.0000000000001401.
7. Gao, X.; Li, B.; Chu, W.; Sun, X.; Sun, C. Alien hand syndrome following corpus callosum infarction: A case report and review of the literature. *Exp. Ther. Med.* **2016**, *12*, 2129–2135, doi:10.3892/etm.2016.3608.
8. Khalighinejad, N.; Kunnumpurath, A.; Bertini, C.; Ladavas, E.; Haggard, P. Subliminal modulation of voluntary action experience: A neuropsychological investigation. *Cortex* **2017**, *90*, 58–70, doi:10.1016/j.cortex.2017.02.012.
9. Mahawish, K. Corpus callosum infarction presenting with anarchic hand syndrome. *BMJ Case Rep.* **2016**, bcr2016216071, doi:10.1136/bcr-2016-216071.
10. Qureshi, I.A.; Korya, D.; Kassar, D.; Moussavi, M. Case Report: 84 year-old woman with alien hand syndrome. *F1000Research* **2016**, *5*, 1564, doi:10.12688/f1000research.9096.1.
11. Abdulla, M.; Saseendran, R. Transient left alien hand syndrome after left anterior cerebral artery infarction. *Ann. Indian Acad. Neurol.* **2019**, *22*, 118, doi:10.4103/aian.AIAN_143_18.

B. DIFFERENCES IN THE FREQUENCY OF AHS SYMPTOMS ASSOCIATED WITH VARIOUS LESION SITES.

Table SM2. Summary of the patients (with associated symptoms) resulting from the integration of data by Moro et al., 2015 and an update of the literature from 2016-2020

	Anterior lesion		Posterior lesion		Mixed		Callosal + Paracallosal		Thalamic
(percentage frequency)	Right AH	Left AH	Right AH	Left AH	Right AH	Left AH	Right AH**	Left AH	Left AH**

**Total cases reviewed**		16	19	6	18	8	10	2	9	2
**(semi) purposeful AH movements**	**Mag apr**	11	10	0	0	2	5	0	0	0
	**Grasping**	12	15	0	6	4	5	0	3	1
	**Forced Grasp**	11	5	1	2	2	2	0	1	0
	**Groping**	13	14	1	4	4	5	0	3	0
	**Other**	5	4	0	1	0	1	0	2	0
**Non purposeful AH movements**	**Expl beh**	0	0	1	1	2	0	0	0	0
	**Rep mov**	4	0	4	3	3	2	0	1	2
	**Self grabbing**	3	1	1	8	4	2	0	2	2
	**Levitation**	0	0	3	5	2	2	0	0	1
	**Noct mov**	1	0	0	2	1	3	0	1	1
**uncontrolled bilateral hand movements**	**Int conf**	9	14	2	5	3	4	2	8	0
	**Diag Dyspraxia**	4	7	2	3	2	3	1	8	0
	**Resp**	1	3	0	4	0	4	0	0	0
	**Mirr, Sink**	2	2	0	1	1	3	0	2	0
**Feelings**	**Alien, Pers**	8	8	2	12	5	2	1	3	2
	**Rest, Aut, Avo**	11	7	4	9	4	3	1	1	1
**Disconnec-tion symptoms**	**Unil apr, agr**	8	15	0	5	1	4	0	2	0
	**Tact agn, Int tran**	5	6	1	6	1	3	0	2	1
**Frequently associated sensorimotor deficits**	**Unresponsiveness**	5	6	0	0	0	4	0	3	0
	**Dexterity**	1	6	2	6	5	2	0	1	2
	**Bi Inc, Tap, Seq**	6	9	4	2	3	2	0	6	0
	**Sensory loss**	0	1	1	9	2	3	0	0	2
	**Lower limb hyp**	5	8	2	5	1	6	0	3	1
	**Mutism (initial)**	5	6	0	0	1	1	0	0	0
**Cognitive deficits**	**Language dis**	8	5	3	1	3	1	0	3	0
	**Verbal mem**	1	1	1	2	1	1	0	1	0
	**Vis mem, Cons**	2	5	2	6	1	2	0	1	0
	**Discalculia**	1	1	1	0	2	1	0	0	0
	**Attentional dis**	2	4	1	4	2	0	0	1	0
	**Neglect**	0	6	3	7	2	4	0	1	2
	**Disexecutive synd**	2	5	0	1	1	0	0	0	0
	**Apraxia**	1	0	1	0	4	1	1	0	0

Table SM3 Frequency of AHS symptoms found in the different subtypes of the syndrome. Data are collected from an analysis of a previous review by Moro et al., 2015, updated with the new review of literature (SM-A) ** Due to the small sample size, statistical analyses for Callosal right anarchic hand and Thalamic left anarchic hand were not included.

**(percentage frequency)**		**Anterior lesion**		**Posterior lesion**		**Mixed**		**Callosal + Paracallosal**		**Thalamic**
		**Right AH**	**Left AH**	**Right AH**	**Left AH**	**Right AH**	**Left AH**	**Right AH**	**Left AH**	**Left AH**
**Total cases reviewed**		16	19	6	18	8	10	2	9	2
**(semi) purposeful**	**Mag apr**	69%	53%	0%	0%	25%	50%	0%	0%	0%
	**Grasping**	75%	79%	0%	33%	50%	50%	0%	33%	50%

**AH movements**	**Forced Grasp**	69%	26%	17%	11%	25%	20%	0%	11%	0%
	**Groping**	81%	74%	17%	22%	50%	50%	0%	33%	0%
	**Other**	31%	21%	0%	6%	0%	10%	0%	22%	0%
**Non purposeful AH movements**	**Expl beh**	0%	0%	17%	6%	25%	0%	0%	0%	0%
	**Rep mov**	25%	0%	67%	17%	38%	20%	0%	11%	100%
	**Self grabbing**	19%	5%	17%	44%	50%	20%	0%	22%	100%
	**Levitation**	0%	0%	50%	28%	25%	20%	0%	0%	50%
	**Noct mov**	6%	0%	0%	11%	12%	30%	0%	11%	50%
**uncontrolled bilateral hand movements**	**Int conf**	56%	74%	33%	28%	38%	40%	100%	89%	0%
	**Diag Dyspraxia**	25%	37%	33%	17%	25%	30%	50%	89%	0%
	**Resp**	6%	16%	0%	22%	0%	40%	0%	0%	0%
	**Mirr, Sink**	13%	11%	0%	6%	12%	30%	0%	22%	0%
**Feelings**	**Alien, Pers**	50%	42%	33%	67%	62%	20%	50%	33%	100%
	**Rest, Aut, Avo**	69%	37%	67%	50%	50%	30%	50%	11%	50%
**Disconnec-tion symptoms**	**Unil apr, agr**	50%	79%	0%	28%	12%	40%	0%	22%	0%
	**Tact agn, Int tran**	31%	32%	17%	33%	12%	30%	0%	22%	50%
**Frequently associated sensorimotor deficits**	**Unresponsiveness**	31%	32%	0%	0%	0%	40%	0%	33%	0%
	**Dexterity**	6%	32%	33%	33%	62%	20%	0%	11%	100%
	**Bi Inc, Tap, Seq**	38%	47%	67%	11%	38%	20%	0%	67%	0%
	**Sensory loss**	0%	5%	17%	50%	25%	30%	0%	0%	100%
	**Lower limb hyp**	31%	42%	33%	27%	12%	60%	0%	33%	50%
	**Mutism (initial)**	31%	32%	0%	0%	12%	10%	0%	0%	0%
**Cognitive deficits**	**Language dis**	50%	26%	50%	6%	38%	10%	0%	33%	0%
	**Verbal mem**	6%	5%	17%	11%	12%	10%	0%	11%	0%
	**Vis mem, Cons**	13%	26%	33%	33%	12%	20%	0%	11%	0%
	**Discalculia**	6%	5%	17%	0%	25%	10%	0%	0%	0%
	**Attentional dis**	13%	21%	17%	22%	25%	0%	0%	11%	0%
	**Neglect**	0%	32%	50%	39%	25%	40%	0%	11%	100%
	**Disexecutive synd**	13%	26%	0%	6%	12%	0%	0%	0%	0%
	**Apraxia**	6%	0%	17%	0%	50%	10%	50%	0%	0%

Table SM4. ***Differences in frequencies of symptoms associated with Anterior or Posterior right hemisphere damage and left anarchic hand in the patients analysed in the review of the literature***. In yellow, symptoms that are more frequent with anterior lesions; in green, symptoms that are more frequent with posterior lesions. Comparisons were made by means of χ^2 tests.

	(percentage frequency)	Anterior<br>Left AH	Posterior<br>Left AH	p value
	**Total cases reviewed**	19	18	
**(semi) purposeful AH movements**	**Mag apr**	10	0	< 0.01
	**Grasping**	15	6	0.01
	**Forced Grasp**	5	2	0.24

	Groping	14	4	< 0.01
	Other	4	1	0.17
Non purposeful AH movements	Expl beh	0	1	0.30
	Rep mov	0	3	0.06
	Self grabbing	1	8	0.01
	Levitation	0	5	0.01
	Noct mov	0	2	0.14
uncontrolled bilateral hand movements	Int conf	14	5	0.01
	Diag Dyspraxia	7	3	0.17
	Resp	3	4	0.62
	Mirr. Sink	2	1	0.58
Feelings	Alien. Pers	8	12	0.13
	Rest. Aut. Avo	7	9	0.42
Disconnec-tion symptoms	Unil apr. agr	15	5	< 0.01
	Tact agn. Int tran	6	6	0.91
Frequently associated sensorimotor deficits	Unresponsiveness	6	0	0.01
	Dexterity	6	6	0.91
	Bi Inc. Tap. Seq	9	2	0.02
	Sensory loss	1	9	< 0.01
	Lower limb hyp	8	5	0.36
	Mutism (initial)	6	0	0.01
Cognitive deficits	Language dis	5	1	0.09
	Verbal mem	1	2	0.51
	Vis mem. Cons	5	6	0.64
	Discalculia	1	0	0.32
	Attentional dis	4	4	0.93
	Neglect	6	7	0.64
	Disexecutive synd	5	1	0.09
	Apraxia	0	0	-

*Table SM5. **Differences in frequencies of symptoms associated with Posterior right hemisphere damage or mixed (anterior and posterior) left anarchic hand in the patients analysed in the review of the literature**.* In yellow, symptoms that are more frequent with posterior lesions; in green, symptoms that are more frequent with callosal lesions. Comparisons were made by means of χ^2 tests.

(percentage frequency)		Posterior Left AH	Mixed Left AH	p value
Total cases reviewed		18	10	
(semi) purposeful	Mag apr	0	5	< 0.01
	Grasping	6	5	0.39

AH movements	Forced Grasp	2	2	0.52
	Groping	4	5	0.13
	Other	1	1	0.66
Non purposeful AH movements	Expl beh	1	0	0.45
	Rep mov	3	2	0.83
	Self grabbing	8	2	0.20
	Levitation	5	2	0.65
	Noct mov	2	3	0.21
uncontrolled bilateral hand movements	Int conf	5	4	0.51
	Diag Dyspraxia	3	3	0.41
	Resp	4	4	0.32
	Mirr, Sink	1	3	0.08
Feelings	Alien, Pers	12	2	0.02
	Rest, Aut, Avo	9	3	0.31
Disconnec-tion symptoms	Unil apr, agr	5	4	0.51
	Tact agn, Int tran	6	3	0.86
Frequently associated sensorimotor deficits	Unresponsiveness	0	4	< 0.01
	Dexterity	6	2	0.45
	Bi Inc, Tap, Seq	2	2	0.52
	Sensory loss	9	3	0.31
	Lower limb hyp	5	6	0.09
	Mutism (initial)	0	1	0.17
Cognitive deficits	Language dis	1	1	0.66
	Verbal mem	2	1	0.93
	Vis mem, Cons	6	2	0.45
	Discalculia	0	1	0.17
	Attentional dis	4	0	0.11
	Neglect	7	4	0.95
	Disexecutive synd	1	0	0.45
	Apraxia	0	1	0.17

Table SM6. ***Differences in frequencies of symptoms associated with Posterior right hemisphere damage or Callosal lesion and left anarchic hand in the patients analysed in the review of the literature***. In yellow, symptoms that are more frequent with posterior lesions; in green, symptoms that are more frequent with callosal lesions. Comparisons were made by means of χ^2 tests.

(percentage frequency)		Posterior Left AH	Callosal Left AH	p value
Total cases reviewed		18	9	
(semi) purposeful AH	Mag apr	0	0	-
	Grasping	6	3	1.00
	Forced Grasp	2	1	1.00

movements	Groping	4	3	0.53
	Other	1	2	0.19
Non purposeful AH movements	Expl beh	1	0	0.47
	Rep mov	3	1	0.70
	Self grabbing	8	2	0.26
	Levitation	5	0	0.08
	Noct mov	2	1	1.00
uncontrolled bilateral hand movements	Int conf	5	8	< 0.01
	Diag Dyspraxia	3	8	< 0.01
	Resp	4	0	0.13
	Mirr, Sink	1	2	0.19
Feelings	Alien, Pers	12	3	0.10
	Rest, Aut, Avo	9	1	0.05
Disconnec-tion symptoms	Unil apr, agr	5	2	0.76
	Tact agn, Int tran	6	2	0.55
Frequently associated sensorimotor deficits	Unresponsiveness	0	3	0.01
	Dexterity	6	1	0.21
	Bi Inc, Tap, Seq	2	6	< 0.01
	Sensory loss	9	0	0.01
	Lower limb hyp	5	3	0.77
	Mutism (initial)	0	0	-
Cognitive deficits	Language dis	1	3	0.06
	Verbal mem	2	1	1.00
	Vis mem, Cons	6	1	0.21
	Discalculia	0	0	-
	Attentional dis	4	1	0.48
	Neglect	7	1	0.14
	Disexecutive synd	1	0	0.47
	Apraxia	0	0	-

Table SM7. ***Differences in frequencies of symptoms associated with Anterior right hemisphere damage or mixed (anterior and posterior) left anarchic hand in the patients analysed in the review of the literature***. In yellow, symptoms that are more frequent with anterior lesions; in green, symptoms that are more frequent with mixed lesions.

(percentage frequency)		Anterior Left AH	Mixed Left AH	p value
Total cases reviewed		19	10	
(semi) purposeful	Mag apr	10	5	0.89
	Grasping	15	5	< 0.01

AH movements	Forced Grasp	5	2	0.71
	Groping	14	5	0.20
	Other	4	1	0.45
Non purposeful AH movements	Expl beh	0	0	-
	Rep mov	0	2	0.04
	Self grabbing	1	2	0.22
	Levitation	0	2	0.04
	Noct mov	0	3	0.01
uncontrolled bilateral hand movements	Int conf	14	4	0.08
	Diag Dyspraxia	7	3	0.71
	Resp	3	4	0.15
	Mirr, Sink	2	3	0.19
Feelings	Alien, Pers	8	2	0.23
	Rest, Aut, Avo	7	3	0.71
Disconnec-tion symptoms	Unil apr, agr	15	4	0.04
	Tact agn, Int tran	6	3	0.93
Frequently associated sensorimotor deficits	Unresponsiveness	6	4	0.65
	Dexterity	6	2	0.51
	Bi Inc, Tap, Seq	9	2	0.15
	Sensory loss	1	3	0.07
	Lower limb hyp	8	6	0.36
	Mutism (initial)	6	1	0.20
Cognitive deficits	Language dis	5	1	0.30
	Verbal mem	1	1	0.63
	Vis mem, Cons	5	2	0.71
	Discalculia	1	1	0.63
	Attentional dis	4	0	0.12
	Neglect	6	4	0.65
	Disexecutive synd	5	0	0.07
	Apraxia	0	1	0.16

Table SM8. ***Differences in frequencies of symptoms associated with Anterior right hemisphere damage or Callosal lesion and left anarchic hand in the patients analysed in the review of the literature***. In yellow, symptoms that are more frequent with anterior lesions; in green, symptoms that are more frequent with callosal lesions.

(percentage frequency)		Anterior Left AH	Callosal Left AH	p value
Total cases reviewed		19	9	
(semi)	Mag apr	10	0	0.01

purposeful AH movements	Grasping	15	3	0.02
	Forced Grasp	5	1	0.36
	Groping	14	3	0.04
	Other	4	2	0.94
Non purposeful AH movements	Expl beh	0	0	-
	Rep mov	0	1	0.14
	Self grabbing	1	2	0.18
	Levitation	0	0	-
	Noct mov	0	1	0.14
uncontrolled bilateral hand movements	Int conf	14	8	0.36
	Diag Dyspraxia	7	8	0.01
	Resp	3	0	0.21
	Mirr, Sink	2	2	0.41
Feelings	Alien, Pers	8	3	0.66
	Rest, Aut, Avo	7	1	0.16
Disconnec-tion symptoms	Unil apr, agr	15	2	< 0.01
	Tact agn, Int tran	6	2	0.61
Frequently associated sensorimotor deficits	Unresponsiveness	6	3	0.93
	Dexterity	6	1	0.24
	Bi Inc, Tap, Seq	9	6	0.34
	Sensory loss	1	0	0.48
	Lower limb hyp	8	3	0.66
	Mutism (initial)	6	0	0.06
Cognitive deficits	Language dis	5	3	0.70
	Verbal mem	1	1	0.57
	Vis mem, Cons	5	1	0.36
	Discalculia	1	0	0.48
	Attentional dis	4	1	0.52
	Neglect	6	1	0.24
	Disexecutive synd	5	0	0.09
	Apraxia	0	0	-

Table SM9. Differences in frequencies of symptoms associated with Anterior left hemisphere damage or Posterior lesion and right anarchic hand in the patients analysed in the review of the literature. In yellow, symptoms that are more frequent with anterior lesions; in green, symptoms that are more frequent with posterior lesions.

(percentage frequency)	Anterior Right AH	Posterior Right AH	p value

Total cases reviewed		16	6	
(semi) purposeful AH movements	Mag apr	11	0	< 0.01
	Grasping	12	0	< 0.01
	Forced Grasp	11	1	0.03
	Groping	13	1	0.01
	Other	5	0	0.12
Non purposeful AH movements	Expl beh	0	1	0.09
	Rep mov	4	4	0.07
	Self grabbing	3	1	0.91
	Levitation	0	3	< 0.01
	Noct mov	1	0	0.53
uncontrolled bilateral hand movements	Int conf	9	2	0.34
	Diag Dyspraxia	4	2	0.70
	Resp	1	0	0.53
	Mirr, Sink	2	0	0.36
Feelings	Alien, Pers	8	2	0.48
	Rest, Aut, Avo	11	4	0.93
Disconnec-tion symptoms	Unil apr, agr	8	0	0.03
	Tact agn, Int tran	5	1	0.49
Frequently associated sensorimotor deficits	Unresponsiveness	5	0	0.12
	Dexterity	1	2	0.10
	Bi Inc, Tap, Seq	6	4	0.22
	Sensory loss	0	1	0.09
	Lower limb hyp	5	2	0.93
	Mutism (initial)	5	0	0.12
Cognitive deficits	Language dis	8	3	1.00
	Verbal mem	1	1	0.45
	Vis mem, Cons	2	2	0.26
	Discalculia	1	1	0.45
	Attentional dis	2	1	0.80
	Neglect	0	3	< 0.01
	Disexecutive synd	2	0	0.36
	Apraxia	1	1	0.45

Table SM10. Differences in frequencies of symptoms associated with Anterior left hemisphere damage or mixed (anterior and posterior) right anarchic hand in the patients analysed in the review of the literature. In yellow, symptoms that are more frequent with anterior lesions; in green, symptoms that are more frequent with mixed lesions.

(percentage frequency)		Anterior Right AH	Mixed Right AH	p value
Total cases reviewed		16	8	
(semi) purposeful AH movements	Mag apr	11	2	0.04
	Grasping	12	4	0.22
	Forced Grasp	11	2	0.04
	Groping	13	4	0.11
	Other	5	0	0.08
Non purposeful AH movements	Expl beh	0	2	0.04
	Rep mov	4	3	0.53
	Self grabbing	3	4	0.11
	Levitation	0	2	0.04
	Noct mov	1	1	0.60
uncontrolled bilateral hand movements	Int conf	9	3	0.39
	Diag Dyspraxia	4	2	1.00
	Resp	1	0	0.47
	Mirr, Sink	2	1	1.00
Feelings	Alien, Pers	8	5	0.56
	Rest, Aut, Avo	11	4	0.37
Disconnec-tion symptoms	Unil apr, agr	8	1	0.07
	Tact agn, Int tran	5	1	0.32
Frequently associated sensorimotor deficits	Unresponsiveness	5	0	0.08
	Dexterity	1	5	< 0.01
	Bi Inc, Tap, Seq	6	3	1.00
	Sensory loss	0	2	0.04
	Lower limb hyp	5	1	0.32
	Mutism (initial)	5	1	0.32
Cognitive deficits	Language dis	8	3	0.56
	Verbal mem	1	1	0.60
	Vis mem, Cons	2	1	1.00
	Discalculia	1	2	0.19
	Attentional dis	2	2	0.44
	Neglect	0	2	0.04
	Disexecutive synd	2	1	1.00
	Apraxia	1	4	0.01

Table SM11. Differences in frequencies of symptoms associated with Posterior left hemisphere damage or mixed (anterior and posterior) right anarchic hand in the patients analysed in the review of the literature. In green, symptoms that are more frequent with mixed lesions.

(percentage frequency)		Posterior Right AH	Mixed Right AH	p value
Total cases reviewed		6	8	
(semi) purposeful AH movements	Mag apr	0	2	0.19
	Grasping	0	4	0.04
	Forced Grasp	1	2	0.71
	Groping	1	4	0.20
	Other	0	0	-
Non purposeful AH movements	Expl beh	1	2	0.71
	Rep mov	4	3	0.28
	Self grabbing	1	4	0.20
	Levitation	3	2	0.33
	Noct mov	0	1	0.37
uncontrolled bilateral hand movements	Int conf	2	3	0.87
	Diag Dyspraxia	2	2	0.73
	Resp	0	0	-
	Mirr, Sink	0	1	0.37
Feelings	Alien, Pers	2	5	0.28
	Rest, Aut, Avo	4	4	0.53
Disconnec-tion symptoms	Unil apr, agr	0	1	0.37
	Tact agn, Int tran	1	1	0.83
Frequently associated sensorimotor deficits	Unresponsiveness	0	0	-
	Dexterity	2	5	0.28
	Bi Inc, Tap, Seq	4	3	0.28
	Sensory loss	1	2	0.71
	Lower limb hyp	2	1	0.35
	Mutism (initial)	0	1	0.37
Cognitive deficits	Language dis	3	3	0.64
	Verbal mem	1	1	0.83
	Vis mem, Cons	2	1	0.35
	Discalculia	1	2	0.71
	Attentional dis	1	2	0.71
	Neglect	3	2	0.33
	Disexecutive synd	0	1	0.37
	Apraxia	1	4	0.20
